# Outcomes of Minimally Invasive and Robot-Assisted Esophagectomy for Esophageal Cancer

**DOI:** 10.3390/cancers14153667

**Published:** 2022-07-28

**Authors:** Kian C. Banks, Diana S. Hsu, Jeffrey B. Velotta

**Affiliations:** 1 Department of Thoracic Surgery, Kaiser Permanente Northern California, Oakland, CA 94611, USA; dianahsu@alamedahealthsystem.org (D.S.H.); jeffrey.b.velotta@kp.org (J.B.V.); 2 Department of Surgery, UCSF East Bay, Oakland, CA 94602, USA

**Keywords:** indications, outcomes, review, minimally invasive esophagectomy, robot-assisted esophagectomy, esophageal cancer, Ivor Lewis, McKeown

## Abstract

**Simple Summary:**

This is an invited review for the special edition, “Minimally Invasive Surgery for Cancer: Indications and Outcomes.” Indications to perform minimally invasive techniques for esophagectomy rather than the classic open technique do not exist. This review outlines the current research by comparing outcomes among minimally invasive esophagectomy, robot-assisted esophagectomy, and open esophagectomy. After determining the benefits of each technique in terms of each outcome, the discussion focuses on how surgeons may use the presented information to determine which approach is most appropriate. We hope this study provides a comprehensive review of the current state of the literature regarding minimally invasive esophagectomy, as well as a guide for surgeons who treat patients with esophageal cancer.

**Abstract:**

With the evolution of minimally invasive esophagectomy (MIE) and robot-assisted minimally invasive esophagectomy (RAMIE), questions remain regarding the benefits and indications of these methods. Given that set indications do not exist, this article aims first to review the reported outcomes of MIE, RAMIE, and open esophagectomy. Then, considerations based on the reported outcomes are discussed to guide surgeons in selecting the best approach. MIE and RAMIE offer the potential to improve outcomes for esophagectomy patients; however, surgeon experience as well as individual patient factors play important roles when deciding upon the surgical approach.

## 1. Introduction/Background

Since minimally invasive esophagectomies (MIEs) were first performed, they have undergone a significant evolution [1,2]. In 1995, DePaula et al. demonstrated their experience in laparoscopic transhiatal esophagectomy techniques, with acceptable short-term outcomes [3]. Three years later, Luketich et al. published their experience using a totally minimally invasive technique including laparoscopy and thoracoscopy, in addition to a cervical incision for the anastomosis [4]. In 2000, Nguyen et al. retrospectively compared MIE to both open transthoracic and transhiatal approaches, with findings of improved operative times, blood loss, and length of stay among the MIE cases [5]. Luketich et al. then expanded upon their growing experience with MIE by publishing outcomes from 222 McKeown MIE cases, including an acceptable anastomotic leak rate of 11% [6]. Just one year later, Kernstine et al. published a case report detailing a three-field robot-assisted minimally invasive esophagectomy (RAMIE) technique with successful subsequent recovery in a patient with esophageal adenocarcinoma. In the subsequent years, groups began to publish promising outcomes from their series of esophageal cancer patients treated with RAMIE [7,8].

As MIE and RAMIE evolved, the need for studies to assess their effectiveness in oncologic resections was clear. Such studies have been performed over the years, and continue to be performed as the landscape evolves. Given that clear indications for minimally invasive techniques do not yet exist, in order to understand the appropriate use for these approaches, it is critical to understand their benefits. This review aims to guide the selection of an operative approach by first detailing the effect of each approach on outcomes of interest after esophagectomy. Once this context is established, indications for minimally invasive approaches are discussed.

## 2. Outcomes

### 2.1. Study Inclusion

In order to compare outcomes across various surgical approaches, studies were carefully selected in a comprehensive manner that prioritized recent high-quality studies. Given the recent adoption of these minimally invasive approaches and their ongoing development, the chosen studies were limited to the last decade, with priority placed on studies from the last few years. Randomized controlled trials were considered the gold-standard, and have been emphasized. Meta-analyses have been included to provide additional supporting evidence. Retrospective studies were included selectively, but propensity score-matched analyses were used when possible. The studies included in the outcome comparisons can be found in Table 1.

The following outcome comparisons are focused on MIE, RAMIE, and open procedures. Though not the primary focus of this review, hybrid MIE is discussed separately in Section 2.13.

The results from the available studies for each outcome are combined to provide readers with an understanding of the range of results that are reported (Table 2). Interpretation of these ranges must be conducted with caution because the combined data do not necessarily come from studies with identical populations or study designs, and the ranges only represent the high and low extremes reported in each category. Additionally, these ranges are only representative of studies included in this review. They are solely included for the purpose of demonstrating the spectrum of outcomes reported within the cited studies.

### 2.2. Operative Duration

Several studies comparing MIE to open esophagectomy over the last decade have found operative times for MIE to be significantly longer [9,12,14,24]. The TIME randomized controlled trial found MIE to be about 30 min longer than open esophagectomy comparison groups (329 min vs. 299 min, *p* = 0.002) [9].

Similarly, studies over the last several years involving RAMIE have found that it was significantly longer than open comparison groups including the randomized controlled trial by van der Sluis et al., which found RAMIE to be nearly one hour longer than open esophagectomy (349 min vs. 296 min, *p* < 0.001) [15,18,26,29]. In comparisons of RAMIE to MIE, some studies found longer operative times in the RAMIE group [16,19,27,28]. However, Jin et al. found similar times between the two groups in their meta-analysis, and, in a recently published randomized controlled trial, Yang et al. actually found RAMIE to be 41 min faster than MIE (204 min vs. 245 min, *p* < 0.001) [17,32].

Over the last decade, reported case times for MIE (237 to 443 min) and RAMIE (204 to 490 min) are wide ranging while reported times for open esophagectomy (295 to 339 min) are much less variable [9,12,15,16,18,19,21,29,31,32]. These findings are likely related to the relatively new nature of MIE, and especially RAMIE, when compared to open esophagectomy. It is particularly noteworthy that the low ends of these ranges suggest that MIE and RAMIE have the potential to require as little, if not less, operative time as the open procedure.

### 2.3. Estimated Blood Loss

When comparing MIE or RAMIE to open esophagectomy in terms of estimated intraoperative blood loss, the minimally invasive strategies appear to have a considerable advantage [9,14,15,18,24,26,29]. These findings hold true in the randomized controlled trial comparing MIE to open esophagectomy (200 mL vs. 475 mL; *p* < 0.001) as well as the one comparing RAMIE to open esophagectomy (120 mL vs. 200 mL, *p* < 0.001) [9,15].

Reports of estimated blood loss in MIE and RAMIE range from 100 to 350 mL and 120 to 331 mL, respectively, while those of estimated blood loss during open esophagectomy range from 200 to 500 mL [9,15,16,18,19,21,29,31,32].

Interestingly, despite the similar ranges of reported estimated blood loss between MIE and RAMIE, several studies comparing the two have found less estimated blood loss among patients undergoing RAMIE [17,22,27,28]. Others found no difference between the two approaches [16,19,26,32]. In the randomized controlled trial performed by Yang et al. comparing MIE to RAMIE, there was no difference in estimated blood loss (200 mL each, *p* = 0.38) [32].

While estimations of blood loss are inherently subjective, the similar ranges reported among MIE and RAMIE along with the results of the randomized controlled trial comparing the two suggest that both allow for similar limitation of blood loss.

### 2.4. R0 Resection and Lymph Node Yield

Among studies comparing MIE to open, RAMIE to open, and MIE to RAMIE, nearly all found no difference in terms of R0 resections [9,17,18,19,21,32]. A 2021 meta-analysis of 60 studies ranging from 2008 through to 2020 (predominantly retrospective) found higher rates of R0 resection among RAMIE compared to MIE (OR 2.84, 95% CI 1.53–5.26), however, this contrasts with results of the more recent randomized controlled trial by Yang et al. that found no difference between RAMIE and MIE in terms of this metric (95.0% vs. 92.1%, respectively; *p* = 0.26) [27,32]. Reports of R0 resections for MIE, RAMIE, and open esophagectomy range from 90.1% to 97.5%, 92% to 96.9%, and 83.9% to 97.2%, respectively [9,18,19,21,31,32,33]. 

In addition to achieving an R0 resection, lymph node yield has been associated with improved overall and disease-free survival among esophagectomy patients [35,36]. Although a meta-analysis by Dantoc et al. from 2012 suggested higher lymph node yield via MIE compared to open esophagectomy, a later meta-analysis of 57 prospective studies by Yibulayin et al. revealed no such difference [10,14]. Similarly, the TIME randomized controlled trial did not find increased lymph node yield in MIE over open (20 lymph nodes vs. 21 lymph nodes, respectively; *p* = 0.85) [9]. 

Studies are limited comparing RAMIE to open esophagectomy in terms of lymph node yield. While the prospective trial by Sarkaria et al. found higher average lymph node yield in RAMIE compared to open esophagectomy (25 and 22 lymph nodes, respectively; *p* = 0.05), the ROBOT randomized controlled trial by van der Sluis et al. did not find such a difference (27 lymph nodes vs. 25 lymph nodes, respectively; *p* = 0.41) [15,18]. In a retrospective study by Faermark et al., MIE was found to have a higher lymph node yield than a hybrid procedure of laparoscopy and thoracotomy (31 lymph nodes vs. 28 lymph nodes, respectively; *p* = 0.04) [33].

When comparing RAMIE and MIE one meta-analysis by Li et al. of 13 retrospective studies found higher lymph node yield among RAMIE while several other studies revealed no difference [16,17,19,22,26,27]. Interestingly, the RAMIE randomized controlled trial by Yang et al. specifically found higher average thoracic lymph node yield in RAMIE compared to MIE among patients who had undergone neoadjuvant therapy (15 lymph nodes vs. 12 lymph nodes, respectively; *p* = 0.016), but not in patients who did not undergo neoadjuvant treatment (14 lymph nodes each; *p* = 0.28) [32].

Reported ranges over the last decade of lymph node yield among MIE, RAMIE, and open esophagectomy are 12 to 23 nodes, 19 to 29 nodes, and 21 to 25 nodes, respectively [9,12,15,16,18,19,21,29,31,32]. It is not entirely clear from this data whether there is a clear benefit of one approach over another in terms of lymph node yield.

### 2.5. Anastomotic Leak

Of all studies reviewed comparing MIE to open esophagectomy, RAMIE to open esophagectomy, and MIE to RAMIE, only a single retrospective study comparing RAMIE to open esophagectomy found a difference in anastomotic leak rate (8.0% vs. 25.3%; *p* = 0.004) [9,12,14,15,18,19,24,26,27,28,29,32]. It is worth noting the particularly high leak rate reported among the open group in this study [29]. Additionally, this study accounted for the highest average estimated blood loss among open esophagectomies of any of the studies reviewed (500 mL), as well as the highest short-term mortality (13.3%) among open esophagectomies.

The ranges of reported anastomotic leak rates among MIE, RAMIE, and open esophagectomy among the reviewed studies were 2.1% to 13.2%, 3.0% to 24.1%, and 7.0% to 25.3%, respectively [9,12,15,18,19,21,29,30,31,32]. Interestingly, the 24.1% leak rate among RAMIE was found in the ROBOT randomized controlled trial which found a similarly high leak rate among open esophagectomy as well (20.0%) [15]. Cervical anastomoses were used for both RAMIE and open esophagectomies in this trial. 

The type and location of anastomosis itself may also play a role in anastomotic leak rate. Many of the studies cited include heterogeneous anastomosis techniques (cervical hand-sewn, transthoracic stapled, etc.). Few randomized controlled trials exist that directly compare anastomotic techniques, and those that have been performed have mixed results [37,38,39].

Based on the results of the reviewed studies, it is difficult to determine a superior operative approach in terms of minimizing anastomotic leak. The lower range of leak rates among MIE and RAMIE are encouraging, however, and suggest that these approaches may ultimately help surgeons limit anastomotic leak rate. Additionally, several of these studies did not differentiate between cervical and intrathoracic anastomosis sites, which is discussed in more detail later (within the “Outcomes Among Operative Techniques” section).

### 2.6. Pulmonary Complications

Several post-operative pulmonary complications such as pneumonia, pleural effusion, empyema, pneumothorax, respiratory failure, and pulmonary embolism are compared across studies, either as individual complications or as total pulmonary complications. The TIME randomized controlled trial found fewer pulmonary infections in the MIE group compared to the open esophagectomy group (12% vs. 34%; *p* = 0.005) [9]. Several meta-analyses have similarly found reduced pulmonary complications among MIE compared to open esophagectomy patients [14,24,25]. Interestingly, a Society of Thoracic Surgeons National Database comparison of early surgical outcomes did not find a difference in pulmonary complications between MIE and open esophagectomy (28.3 vs. 25.7%; *p* = 0.15) [12].

In comparisons of RAMIE vs. open esophagectomy, studies including the ROBOT trial (32% vs. 58%; *p* = 0.005) have consistently found RAMIE to have lower rates of overall pulmonary complications [15,18,26,29]. Although a few studies comparing pulmonary complications of RAMIE vs. MIE suggest lower rates for RAMIE, the RAMIE trial found no difference between the two (13.8% vs. 14.7%, respectively; *p* = 0.81) [26,27,28,32].

Overall pulmonary complication rates across the included studies ranged from 9.9% to 28.3% for MIE, 13.8 to 32.0% for RAMIE, and 25.7% to 58.0% for open esophagectomy [9,12,15,18,21,29,31,32]. These ranges must be interpreted with additional caution, however, considering the potential variability in each study’s definition of overall pulmonary complications.

### 2.7. Pain

While post-operative pain can be a challenging metric to measure and compare, it would seem to be a reasonable assumption that surgical approaches that avoid use of a thoracotomy may lead to improved post-operative pain control for patients. Results of the TIME trial suggest that MIE does in fact cause less post-operative pain than open esophagectomy according to both a visual analog pain score (10-day linear mixed model with averages of 3/10 and 2/10, respectively; *p* = 0.001) as well as questionnaire responses [9].

The ROBOT trial found reduced pain among patients who underwent RAMIE compared to open esophagectomy using a visual analog pain scale (14-day linear mixed model with averages of 1.9/10 and 2.6/10, respectively; *p* < 0.001), and Sarkaria et al. found a similar reduction in pain severity scores among RAMIE vs. open esophagectomy [15,18]. Evidence comparing pain between RAMIE and MIE is lacking.

### 2.8. Hospital Length of Stay

While hospital length of stay may be affected by other factors, such as enhanced recovery pathways and ability to maintain close outpatient communication, it still serves as a surrogate measurement for factors such as adequate pain control, nutritional independence, and general complexity of patients’ post-operative courses.

Several studies have found significant reductions in hospital length of stay among patients undergoing MIE relative to open esophagectomy [12,14,24]. The TIME trial similarly found a reduction in hospital length of stay among the MIE group (11 days vs. 14 days; *p* = 0.04) [9]. While there is some evidence for a reduction in hospital length of stay among patients undergoing RAMIE compared to open esophagectomy, the ROBOT trial did not find a significant difference between them (14 days and 16 days, respectively; *p* = 0.33) [15,18,29]. Studies comparing MIE to RAMIE, including the RAMIE trial (9 days each, *p* = 0.31), have failed to find a difference in length of stay between the two surgical approaches [16,17,19,27,32]. 

Reported hospital lengths of stay from the analyzed studies range from 3.0 days to 12.5 days for MIE, 9.0 days to 14.0 days for RAMIE, and 10.0 days to 20.0 days for open esophagectomy [9,12,15,16,18,19,21,29,30,31,32]. Though the explanation may be multifactorial, MIE and RAMIE appear to show promise in terms of the time it takes for patients to be ready for discharge post-operatively.

### 2.9. Short-Term Mortality

Studies on MIE and RAMIE generally report short-term mortality as one or more of inpatient mortality, 30-day mortality, 60-day mortality, or 90-day mortality. In attempt to make them as comparable as possible, the longest term recorded mortality within 90 days for each study is used in this discussion and will be referred to as short-term mortality. 

While the majority of studies including the TIME trial do not find a difference in short-term mortality between MIE and open esophagectomy, Yibulayin et al. do note a small but significantly reduced mortality among MIE patients (3.8% vs. 4.5%; *p* < 0.05) in their meta-analysis [9,12,14,25]. 

In comparisons of RAMIE with open esophagectomy, short-term mortality was only lower in RAMIE in a single retrospective study which reported a particularly high short-term mortality for open esophagectomy of 13.3% (4.0% for MIE) [29]. Otherwise, no difference in mortality was found between RAMIE and open esophagectomy including both the prospective study by Sarkaria et al. (2% vs. 4%, respectively; *p* = 0.85) as well as the ROBOT trial (9% vs. 2%, respectively; *p* = 0.11) [15,18]. None of the included studies found a difference in short-term mortality between MIE and RAMIE [16,17,19,26,27,28,32]. 

Short-term mortality for MIE, RAMIE, and open esophagectomy ranged from 0% to 3.8%, 0% to 9%, and 2.0% to 13.3%, respectively, among the included studies [9,12,14,15,16,18,19,21,29,30,31,32]. Based on these results, it appears each of the three surgical approaches can achieve similarly low short-term mortality rates.

### 2.10. Long-Term Survival

Evidence is limited regarding long-term survival. A retrospective study by Burdall et al. suggests that MIE is associated with improved survival, and Ashiku et al. report a median overall survival of 4.6 years among MIE patients [11,31]. The meta-analysis by Guo et al. suggests improved overall survival in MIE compared to open esophagectomy at two years but not five years [13]. More recent meta-analyses suggest no difference in overall survival or disease-free survival at either three years or five years [20,24]. Similarly, the follow up to the TIME trial found no difference between MIE and open esophagectomy in overall survival (50.5% vs. 40.4%, respectively; *p* = 0.21) or disease-free survival (40.2% vs. 35.9%, respectively; *p* = 0.60)) at three years [40]. Even less data is available for survival after RAMIE; however, a follow-up to the ROBOT trial found RAMIE and open esophagectomy to have similar overall survival (41% vs. 40%, respectively; *p* = 0.83) and disease-free survival (42% vs. 43%, respectively; *p* = 0.75) at five years [41].

### 2.11. Hiatal Herniation

Hiatal herniation after esophagectomy is a challenging problem that can be life-threatening. While this complication is generally considered rare, studies have examined whether MIE is associated with increased rates. In a meta-analysis by Murad et al., higher rates of hiatal herniation were found in MIE compared to open esophagectomy (6.5% vs. 2.4%) [23]. A subsequent meta-analysis by Lee et al. found a similar difference (6.0% vs. 3.2%) [34]. However, the true rate of herniation may be higher. Lung et al. retrospectively examined cross-sectional imaging of patients after MIE and found evidence of hiatal herniation in 15% of these patients (64% of which were asymptomatic) [42]. It appears that, with the transition to MIE, rates of hiatal herniation will increase. However, the implications of this complication, and the decision of whether to intervene on asymptomatic cases, remain undetermined.

### 2.12. Outcomes among Operative Techniques

Many studies comparing MIE, RAMIE, and open esophagectomy do not focus on the specific surgical technique used. In fact, some include a heterogeneous mix of techniques. It is therefore important to understand the differences between outcomes of various operative techniques. While there are dozens of variations of techniques that may be used, we focus primarily on the McKeown (consisting of abdominal, thoracic, and cervical operative fields with the anastomosis performed in the cervical region) and Ivor Lewis (consisting of abdominal and thoracic operative fields with an intrathoracic anastomosis) techniques for esophagectomy. We also briefly discuss outcomes of the transhiatal approach (later in this section).

In 2012, in a retrospective analysis of outcomes after MIE in nearly 1000 patients, Luketich et al. compared outcomes between those undergoing Ivor Lewis MIE and McKeown MIE [43]. They found reduced rates of vocal cord paralysis (1% vs. 8%; *p* < 0.001) and acute respiratory distress syndrome (2% vs. 4%; *p* = 0.03) in patients treated using the Ivor Lewis technique compared to the McKeown technique, but they did not find any differences in pulmonary infections, anastomotic leak, or mortality [43].

Deng et al. found much more imbalanced outcomes between McKeown MIE and Ivor Lewis MIE in their meta-analysis of 14 studies (mix of retrospective and prospective cohort studies) [44]. They found McKeown MIE to have more blood loss (weighted mean difference (WMD) 16.9, 95% confidence interval (CI) 3.22–30.58; *p* = 0.02), longer operating times (WMD =36.49, 95% CI =7.12–65.86, *p* = 0.01), longer hospital stays (WMD 1.29, 95% CI 0.27–2.31; *p* = 0.01), more strictures (Odds ratio (OR) 2.07, 95% CI 1.05–4.07; *p* = 0.04), more vocal cord injuries (OR 5.62, 95% CI 3.46–9.14; *p* < 0.001), higher incidence of pulmonary complications (OR 1.96, 95% CI 1.28–3.00; *p* = 0.002), and higher anastomotic leak rates (OR 2.55, 95% CI 1.40–4.63; *p* = 0.002) [44]. 

Wang et al. then performed a meta-analysis of 23 cohort studies, and found that although McKeown MIE was superior in terms of hospital cost (WMD −0.40, 95% CI −0.74 to 0.07; *p* = 0.02), it was inferior in terms of operating time (WMD 23.69, 95% CI 6.26–41.12; *p* = 0.008), hospital length of stay (WMD 1.13, 95% CI 0.45–1.82; *p* = 0.001), recurrent laryngeal nerve injury (OR 5.63, 95% CI 3.99–7.94; *p* < 0.001), pulmonary complications (OR 1.89, 95% CI 1.54–2.32; *p* < 0.001), anastomotic stenosis (OR 2.89, 95% CI 1.97–4.24; *p* < 0.001), anastomotic leak rate (OR 2.97, 95% CI 2.34–3.77; *p* < 0.001), and short-term mortality (OR 2.85, 95% CI 1.55–5.23, *p* < 0.001) [45].

In 2021, Van Workum et al. performed a randomized controlled trial comparing MIE with cervical vs. intrathoracic anastomosis [46]. They found that, although there were no differences in mortality, MIE with intrathoracic anastomosis had superior hospital length of stay (10.0 days vs. 11.5 days; *p* = 0.003), rate of recurrent laryngeal nerve injury (0% vs. 7.3%; *p* = 0.003), anastomotic leak rate (12.3% vs. 34.1%; *p* < 0.001), severe complication rate (10.7% vs. 22.0%; *p* = 0.02), and better quality of life in three recorded subdomains (mean differences: dysphagia (−12.2, 95% CI −19.6 to −4.7), choking when swallowing (−10.3, 95% CI −16.4 to 4.2), trouble talking (−15.3 95% CI −22.9 to −7.7)) [46]. 

Given the building evidence in favor of Ivor Lewis MIE, it is important to understand the technique used in the studies included in this review. Of the included randomized controlled trials, the TIME trial included a mix of McKeown and Ivor Lewis techniques, the ROBOT trial included entirely cervical anastomoses, and the RAMIE trial included entirely cervical anastomoses [9,15,32]. While these randomized controlled trials have provided useful information, further trials will be of interest with focus on the Ivor Lewis technique.

While transhiatal MIE gained initial traction, concerns began to grow regarding long-term survival rates, and this technique was largely replaced by the McKeown and Ivor Lewis techniques [2,47]. Recent studies do exist, however, that use techniques such as mediastinoscopy to reassess safety and efficacy of minimally invasive transhiatal esophagectomy [48,49].

### 2.13. Hybrid Minimally Invasive Esophagectomy

While not included in the outcomes discussion, an alternative approach includes a hybrid MIE in which laparoscopy is performed for the abdominal portion of the operation, and a thoracotomy is performed for the thoracic portion. The MIRO randomized controlled trial compared this approach (with intrathoracic anastomosis) to the open approach, and found reduced overall and pulmonary complications in the hybrid approach, with similar three-year survival [50]. A five-year follow-up then confirmed no difference in overall survival, disease-free survival, or recurrence rates [51]. While the role of hybrid MIE remains unclear, in light of the promise shown by totally MIE and RAMIE approaches, further investigation may help determine whether the hybrid approach is a comparable alternative or perhaps an option in cases in which totally minimally invasive approaches are not determined to be feasible.

## 3. Indications

While the National Comprehensive Cancer Network Guidelines provide treatment recommendations for esophageal and esophagogastric junction cancers, including the role of esophagectomy, these outlines do not specify whether to perform open esophagectomy, MIE, or RAMIE [52]. In fact, clearly defined indications for each approach do not exist. Therefore, when the decision to pursue esophagectomy for cancer in an appropriate surgical candidate is made, each surgeon must carefully consider various factors to determine the best approach. 

One fundamental consideration is surgeon experience. As discussed, there are wide ranging reports in the literature among several MIE and RAMIE outcomes. As MIE began gaining traction, Levy et al. emphasized the steep learning curve for this operation as they documented outcomes during their transition to Ivor Lewis MIE [53]. They specifically discussed using mini-thoracotomy as a transition step prior to performing the intrathoracic anastomosis in a minimally invasive fashion [53]. Studies have since suggested that the learning curve for MIE requires at least 35–40 cases of experience to reach proficiency, while van Workum et al. provide evidence that it may take closer to 119 cases before improvement in critical outcomes reach a plateau [54,55,56,57]. Similarly, studies assessing the learning curve in RAMIE suggest that at least 40–60 cases, possibly more, are required [57,58,59]. Regardless of the precise number of cases, these studies suggest the importance of a learning environment that allows gradual autonomy in performing these operations while simultaneously tracking outcomes.

Our group has studied the effects of regionalization of thoracic surgery to higher-volume centers with more experienced surgeons, and has found that regionalization not only reduced length of stay by 2.3 days (95% CI -3.4 to -1.2) and the overall complication rate from 50.7% to 30.2% (OR 0.45, 95% CI 0.25, 0.79), but also long-term outcomes such as one-year survival (OR 0.54, 95% CI 0.29, 1.00) [60,61].

As highlighted in the National Comprehensive Cancer Network Guidelines, appropriate selection of surgical candidates is critical in the evaluation of esophagectomy [52]. While standard considerations apply, Sugita et al. specifically provide evidence that MIE is feasible for patients 75 years of age and older [62]. They found that these patients had comparable disease-free survival to younger patients after propensity score matching [59].

Additional considerations may include tumor location or size. Some have expressed concern that proximal esophageal tumors may not be amenable to Ivor Lewis esophagectomy due to risk of a positive proximal margin [63]. Others have suggested that RAMIE may be of particular benefit for such proximal tumors due to the degrees of freedom afforded by the articulating instrumentation [64]. Similarly, these authors have presented evidence for feasibility of radical resection of select T4b tumors via RAMIE, though long-term outcomes are not available [64].

Ultimately, for cases in which esophagectomy is indicated, MIE and/or RAMIE are likely preferable, assuming the surgeon is adequately experienced in such approaches, and that the location/features of the tumor are amenable to the chosen approach. 

## 4. Future Directions

As technology and experience improve, surgeons must refer to the expanding literature to determine the best approach in a given case. Several trials are in progress that will provide further understanding of the benefits of each approach. Long-term survival data is being collected for the RAMIE trial, and a separate randomized controlled trial, the ROBOT 2 trial, is being conducted to compare RAMIE to MIE using the Ivor Lewis technique rather than the McKeown technique [32,65]. This study also aims to compare cost-effectiveness between the approaches. Similarly, the REVATE randomized controlled trial is ongoing to determine differences between RAMIE and MIE in terms of lymph node harvest [66]. The ROMIO randomized controlled trial aims to shed light on the role of hybrid MIE as it compares open esophagectomy vs. hybrid MIE vs. MIE [67].

## 5. Conclusions

With the ongoing evolution of minimally invasive techniques in esophagectomy, there is promise for improved outcomes and quality of life among patients with esophageal cancer. While it is not clear whether MIE or RAMIE is the superior approach, each is likely superior to open esophagectomy in terms of blood loss, pulmonary complications, pain, and hospital length of stay. It is not yet clear if any one approach shows a benefit in terms of long-term survival. Regardless of the approach used, it is critical for surgeons to be trained within a structured system to preserve patient outcomes while developing the experience level necessary to operate independently. Ultimately, in experienced hands, minimally invasive approaches in esophagectomy are preferable to open esophagectomy in technically feasible cancer cases.

## Figures and Tables

**Table 1:** Studies included in review of outcomes.

Authors	Year	Study Type	Study Size	Study Aim	Main Findings
Biere et al. [9]	2012	RCT	115	Compare Outcomes of MIE vs. Open Esophagectomy	MIE had less pain, fewer pulmonary complications, shorter LOS, less EBL, and similar lymph node yield and mortality
Dantoc et al. [10]	2012	Meta-Analysis	1212	Compare Outcomes of MIE vs. Open Esophagectomy	MIE had better lymph node harvest, but no difference in survival
Burdall et al. [11]	2015	Retrospective Cohort	334	Compare Outcomes of MIE vs. Open Esophagectomy	MIE had better long-term survival
Sihag et al. [12]	2016	Retrospective Database	3780	Compare Outcomes of MIE vs. Open Esophagectomy	MIE had longer operative time and reoperation rates, but shorter LOS and less wound infections
Guo et al. [13]	2016	Meta-Analysis	1549	Compare Outcomes of MIE vs. Open Esophagectomy	MIE had reduced wound infection rates, pulmonary complications, EBL, and better 2-yr survival
Yibulayin et al. [14]	2016	Meta-Analysis	15,790	Compare Outcomes of MIE vs. Open Esophagectomy	MIE had reduced EBL, LOS, pulmonary complications, and short-term mortality
van der Sluis et al. [15]	2019	RCT	112	Compare Outcomes of RAMIE vs. Open Esophagectomy	RAMIE had reduced EBL, pulmonary complications, and pain with similar mortality, but longer operative time
Zhang et al. [16]	2019	Retrospective Cohort	132	Compare Outcomes of MIE vs. RAMIE	RAMIE had longer operative time but similar EBL, LOS, lymph node yield, and short-term mortality
Jin et al. [17]	2019	Meta-Analysis	1862	Compare Outcomes of MIE vs. RAMIE	RAMIE had reduced EBL and vocal cord palsy, but no difference in lymph node yield, short-term mortality, or LOS
Sarkaria et al. [18]	2019	Prospective Cohort	106	Compare Outcomes of RAMIE vs. Open Esophagectomy	RAMIE had reduced pain, EBL, LOS, and pulm complications with increased lymph node yield and operative time
Tagkalos et al. [19]	2020	Retrospective Cohort	100	Compare Outcomes of MIE vs. RAMIE	RAMIE had longer operative times and otherwise similar outcomes
Patel et al. [20]	2020	Meta-Analysis	949	Compare Outcomes of MIE vs. Open Esophagectomy	No difference in overall or disease-free survival
van der Sluis et al. [21]	2021	Prospective Cohort	100	Report Outcomes of RAMIE	Outcomes after RAMIE: 8% anastomotic leak, 17% pulmonary complications, 3% short-term mortality
Li et al. [22]	2021	Meta-Analysis	1749	Compare Outcomes of MIE vs. RAMIE	RAMIE had higher lymph node yield, with reduced EBL and vocal cord palsy
Murad et al. [23]	2021	Meta-Analysis	7943	Compare Outcomes of MIE vs. Open Esophagectomy	MIE had higher rate of hiatal herniation
Müller-Stich et al. [24]	2021	Meta-Analysis	822	Compare Outcomes of MIE vs. Open Esophagectomy	MIE had fewer pulmonary infections, less EBL, shorter LOS, and similar overall and disease-free survival
Coelho et al. [25]	2021	Meta-Analysis	34,465	Compare Outcomes of MIE vs. Open Esophagectomy	MIE had fewer pulmonary complications with similar reoperation, vocal cord palsy, and mortality rates
Mederos et al. [26]	2021	Meta-Analysis	9355	Compare Outcomes of MIE vs. RAMIE vs. Open Esophagectomy	RAMIE had fewer pulmonary complications, but longer operative times and otherwise similar short-term outcomes
Angeramo et al. [27]	2021	Meta-Analysis	6249	Compare Outcomes of MIE vs. RAMIE	RAMIE had longer operative times, but less EBL and pneumonia with higher R0 resection rate
Huang et al. [28]	2021	Meta-Analysis	3838	Compare Outcomes of MIE vs. RAMIE	RAMIE had longer operative times, but less EBL and pulmonary complications.
Merboth et al. [29]	2021	Retrospective Cohort	150	Compare Outcomes of RAMIE vs. Open Esophagectomy	RAMIE had longer operative times, but reduced EBL, anastomotic leaks, pulm complications, LOS, and mortality
Casas et al. [30]	2022	Meta-Analysis	5619	Report Outcomes of MIE	Outcomes after MIE: including 8% anastomotic leaks, 11.2-day LOS, and 2% short-term mortality
Ashiku et al. [31]	2022	Retrospective Cohort	142	Report Outcomes of MIE	Outcomes after MIE: 2.1% anastomotic leaks, 3-day LOS, 9.9% pulm complications, 2.1% mortality, and 4.6-yr survival
Yang et al. [32]	2022	RCT	362	Compare Outcomes of MIE vs. RAMIE	RAMIE had shorter operative time but otherwise similar outcomes
Faermark et al. [33]	2022	Retrospective Cohort	240	Compare Outcomes of MIE vs. Open Esophagectomy	MIE had higher lymph node harvest with similar R0 resection rate
Lee et al. [34]	2022	Meta-Analysis	17,052	Compare Outcomes of MIE vs. Open Esophagectomy	MIE had higher rate of hiatal herniation

RCT, randomized controlled trial; MIE, minimally invasive esophagectomy; RAMIE, robot-assisted minimally invasive esophagectomy; LOS, hospital length of stay; EBL, estimated blood loss; pulm, pulmonary; yr, years.

**Table 2:** Ranges of reported perioperative outcomes by surgical approach.

Outcome	MIE	RAMIE	Open Esophagectomy
Operative Duration, min	237–443	204–490	295–339
Estimated Blood Loss, mL	100–350	120–331	200–500
R0 Resection, %	90.1–97.5	92.0–96.9	83.9–97.2
Lymph Node Yield, *N*	12–23	19–29	21–25
Anastomotic Leak, %	2.1–13.2	3.0–24.1	7.0–25.3
Pulmonary Complications, %	9.9–28.3	13.8–32.0	25.7–58.0
Hospital Length of Stay, days	3.0–12.5	9.0–14.0	10.0–20.0
Short-Term Mortality, %	0–3.8	0–9.0	2.0–13.3

MIE, minimally invasive esophagectomy; RAMIE, robot-assisted minimally invasive esophagectomy; min, minutes; mL, milliliters. Notably, ranges only represent the high and low extremes reported in each category and solely demonstrate the spectrum of outcomes reported within the cited studies.
